# Effect of visit-to-visit blood pressure variability on mild cognitive impairment and probable dementia in hypertensive patients receiving standard and intensive blood pressure treatment

**DOI:** 10.3389/fcvm.2023.1166554

**Published:** 2023-04-17

**Authors:** Hang Guo, Yi Tan, Zhizheng Yao, Zilu Zhang, Jiafu Yan, Xiaofeng Meng

**Affiliations:** ^1^ Department of Education, Beijing Anzhen Hospital, Capital Medical University, Beijing, China; ^2^ Department of Education, Beijing Chaoyang Hospital, Capital Medical University, Beijing, China; ^3^ Department of Cardiology, Affiliated Hospital of Yangzhou University, Yangzhou University, Yangzhou, China; ^4^ Department of Cardiology, Aviation General Hospital, Beijing, China

**Keywords:** systolic blood pressure variability, diastolic blood pressure variability, pulse pressure variability, mild cognitive impairment, probable dementia

## Abstract

**Background:**

High visit-to-visit blood pressure variability (BPV) and hypertension are risk factors for mild cognitive impairment (MCI) and probable dementia (PD). Few articles assessed the effect of BPV on the MCI and PD in intensive blood pressure treatment and the different functions of three types of visit-to-visit BPV: systolic blood pressure variability (SBPV), diastolic blood pressure variability (DBPV) and pulse pressure variability (PPV).

**Methods:**

We performed a *post hoc* analysis of the SPRINT MIND trial. The primary outcomes were MCI and PD. BPV was measured by average real variability (ARV). The Kaplan-Meier curves were used to clarify the difference in tertiles of BPV. We fit Cox proportional hazards models to our outcome. We also did an interaction analysis between the intensive and standard groups.

**Results:**

We enrolled 8,346 patients in the SPRINT MIND trial. The incidence of MCI and PD in the intensive group was lower than that in the standard group. 353 patients had MCI and 101 patients had PD in the standard group while 285 patients had MCI and 75 patients had PD in the intensive group. Tertiles with higher SBPV, DBPV and PPV in the standard group had a higher risk of MCI and PD (all *p* < 0.05). Meanwhile, higher SBPV and PPV in the intensive group were associated with an increased risk of PD (SBPV: HR(95%) = 2.1 (1.1–3.9), *p* = 0.026; PPV: HR(95%) = 2.0 (1.1–3.8), *p* = 0.025 in model 3) and higher SBPV in the intensive group was associated with an increased risk of MCI(HR(95%) = 1.4 (1.2–1.8), *p* < 0.001 in model 3). The difference between intensive and standard blood pressure treatment was not statistically significant when we considered the effect of the higher BPV on the risk of MCI and PD (all *p* for interaction >0.05).

**Conclusion:**

In this *post hoc* analysis of the SPRINT MIND trial, we found that higher SBPV and PPV were associated with an increased risk of PD in the intensive group, and higher SBPV was associated with an increased risk of MCI in the intensive group. The effect of higher BPV on the risk of MCI and PD was not significantly different in intensive and standard blood pressure treatment. These findings emphasized the need for clinical work to monitor BPV in intensive blood pressure treatment.

## 1. Introduction

An estimated 6.5 million Americans age 65 and older are living with Alzheimer's dementia (AD) in 2022. This number could grow to 13.8 million by 2060, barring the development of medical breakthroughs to prevent, slow or cure AD (1). Mild cognitive impairment (MCI) represented the transitional stage from the cognitive changes of normal aging to very early dementia (2) and was suggested as an individual risk factor for subsequent AD with an estimated conversion rate of 10%–15% per year (3).

Visit-to-visit blood pressure variability (BPV) represents a dynamic and characteristic physiologic feature of the cardiovascular system function (4) and is a monitoring marker for patients with hypertension (5). Systolic blood pressure variability (SBPV), diastolic blood pressure variability (DBPV) and pulse pressure variability (PPV) are three different types of visit-to-visit BPV. A meta-analysis of longitudinal studies has identified a dose-response relationship between SBPV and dementia and a long-term plan for reducing SBPV can be a target for preventing mild cognitive impairment (MCI) or probabel dementia (PD) (6). Previous articles also considered DBPV as a key risk marker for cognition decline in later life and several meta-analyses have demonstrated the association between higher DBPV and PD (7–9). However, PPV was not included among the main cognition decline risk factors because of its interdependence with other risk factors and of its dynamic nature (10).

The SPRINT-MIND trial has demonstrated that compared with standard blood pressure treatment (SBP target <140 mm Hg), intensive blood pressure (SBP target <120 mm Hg) can significantly reduce the occurrence of MCI as well as the combined occurrence of MCI or PD (11). The effect of three types of visit-to-visit BPV on the risk of MCI and PD is also unclear when take the intensive blood pressure treatment into account. The aim of the study is thus to assess the association of SBPV, DBPV and PPV with MCI and PD in both standard and intensive blood pressure treatment.

## 2. Methods

### 2.1. Study population

We performed a *post hoc* analysis of the SPRINT (Systolic Blood Pressure Intervention Trial) MIND (Memory and Cognition in Decreased Hypertension) trial. Data were obtained from the National Institutes of Health Biologic Specimen and Data Repository Information Coordinating Center (https://biolincc.nhlbi.nih.gov/studies/sprint/).

The design, rationale, protocols, and main results of the SPRINT MIND trial have been published previously (12, 13). The SPRINT MIND trial was conducted in 102 clinical sites in the United States and enrolled 9,361 participants[mean age, 67.9 years; 3,332 women (35.6%)]. All participants had no diagnosis of dementia, prevalent diabetes mellitus, or history of stroke. Participants were randomly assigned to a systolic blood pressure target of either less than 140 mm Hg (the standard group) or less than 120 mm Hg (the intensive group). The purpose of the SPRINT MIND trial was to describe the effect of intensive blood pressure treatment on the rate of MCI and PD compared with standard blood pressure treatment. In our study, we defined the exposure period as the first 600 days of the SPRINT MIND trial. We used the data of blood pressure measured during the exposure period to calculate the BPV. This was consistent with the previous *post hoc* analyses of the SPRINT MIND trial (14). Moreover, we could ensure the sample size since no patients had MCI or PD in the first 600 days. The outcomes were recorded during the subsequent SPRINT MIND follow-up period.

**Figure 1:**
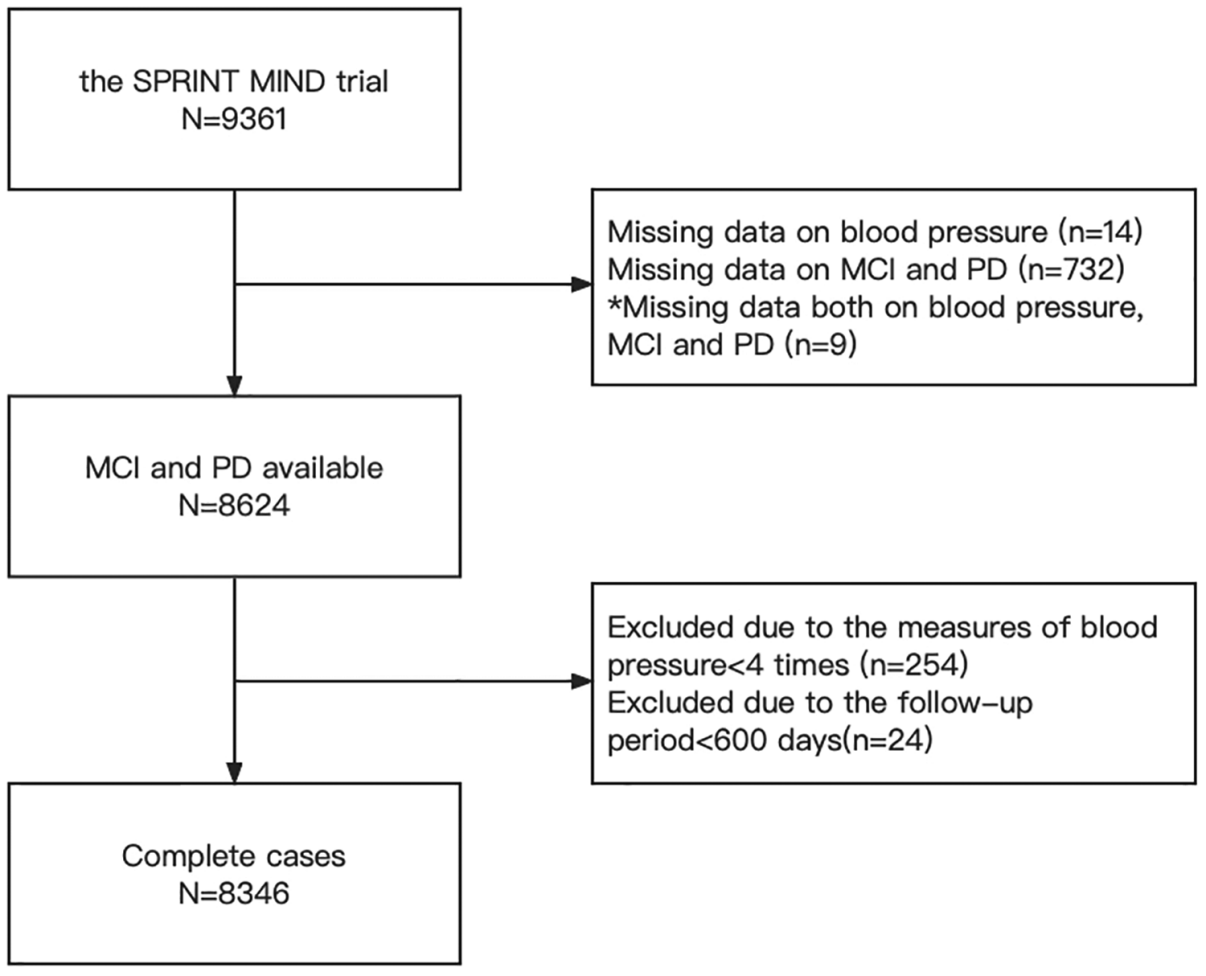
Flowchart of participants’ selection.

Figure 1 shows the delineation of our study population. 9,361 participants were included in this study, while we excluded 737 participants who had missing records of blood pressure and MCI or PD. Among these participants, there were 14 patients without the records of blood pressure, 732 patients without the records of MCI and PD, and 9 patients without all of the records of blood pressure, MCI and PD. In addition, 254 participants were excluded because their blood pressure records were less than 4 times and 24 participants were excluded because the follow-up days were less than 600 days. At last, 8,346 participants were enrolled.

### 2.2. Assessment of BPV

Systolic blood pressure (SBP) and diastolic blood pressure (DBP) were measured at baseline, monthly for the first 3 months and every 3 months thereafter. The mean counts of blood pressure measurement was 10.8 ± 2.5. Pulse pressure (PP) was calculated as follows: PP = SBP − DBP. Mena et al. (15) concluded that the average real variability (ARV) index had a greater predictive value than the standard deviation (SD) and was more useful for determining therapeutic measures aimed at the treatment of BPV. So, we used ARV for blood pressure records with at least four follow-up data to assess the systolic blood pressure variability (SBPV), diastolic blood pressure variability (DBPV) and pulse pressure variability (PPV). Meanwhile, the statistical results measured by SD were also showed in Supplementary Tables S1, S2. Besides, MMD (the difference of maximum minus minimum) meant the delta in blood pressure and Michiaki Nagai have demonstrated the association between MMD and cardiovascular disease (16, 17). So, we also included the statistical results measured by MMD in Supplementary Tables S3, S4. ARV represented the average of absolute differences between successive types and was calculated using the following formula:

$$\mathrm{ARV}=\frac{1}{N-1}\sum_{k=1}^{N-1}\left|BP_{k+1}-BP_{k}\right|$$

where *N* denotes the number of valid *BP* types, and *k* is the order of types.

### 2.3. Assessment of cognitive impairment

The outcomes of this study were probable dementia (PD) and mild cognitive impairment (MCI). All participants were classified into 3 categories: no cognitive impairment, mild cognitive impairment, or probable dementia. MCI represents the transitional stage from the cognitive changes of normal aging to very early dementia (4). The diagnoses of MCI and PD were using standardized diagnostic criteria by 2 adjudicators independently (18, 19). The SPRINT MIND trial followed a 3-step process to ascertain the cognitive status among global cognitive function, learning and memory and processing speed (6). The critical criteria that distinguish MCI from dementia were the preservation of independence in functional abilities (ADLs and IADLs) and lack of significant impairment in social or occupational functioning (20).

### 2.4. Statistical analysis

We used frequencies and percentages to reflect the categorical variables and means ± standard deviations or medians (intertertile ranges) to express the continuous variables based on the data distribution. Chi-square analysis was used to evaluate the differences in categorical variables. We can identify any significant differences between groups *via* the two-tailed *t*-test (normal distribution) or the Mann–Whitney *U* test (skewed distribution). The Kolmogorov–Smirnov test assessed the normal distribution of data.

The Kaplan-Meier curves were used to indicate the difference in the risk of MCI and PD among different BPV tertiles separately and the logrank test was used for comparison between different Kaplan-Meier curves. Cox proportional hazards models were used to describe the effect of SBPV, DBPV or PPV on the rate of MCI and PD. We used a variety of models: Model 1 adjusted age, BMI, sex and race. Model 2 adjusted age, BMI, sex, race, smoke, estimated glomerular filtration rate, Framingham 10-year cardiovascular disease risk score, subclinical cardiovascular disease and total cholesterol. Model 3 further adjusted the mean blood pressure (SBP, DBP or PP) based on the adjustment of Model 2. All models met the proportional hazard assumption. We also performed interaction analyses between different blood pressure treatment arms (standard blood pressure treatment vs. intensive blood pressure treatment). All analyses were performed using the statistical software package R (The R Foundation; http://www.R-project.org). Statistical significance was set at *p* < 0.05.

## 3. Results

### 3.1. Baseline characteristics of the study population

8,346 patients were eventually enrolled in the analysis, including 4,170 in the intensive blood pressure treatment group (the intensive group) and 4,176 in the standard blood pressure treatment group (the standard group). The average age of the participants was 67.7 ± 9.2 years and there were 2,926 (35.1%) women in the study. Supplementary Figures S1, S2 demonstrated the frequency and intervals of BP measurements during the exposure period. As shown in Table 1, the visit-to-visit SBP, DBP and PP of the intensive group were significantly lower than those of the standard group (all *p* < 0.05). Notably, SBPV, DBPV and PPV in the intensive group were also significantly lower than that in the standard group (SBPV: 12.4 ± 5.2 mmHg vs. 11.5 ± 4.9 mmHg, *p* < 0.001; DBPV: 7.4 ± 2.8 mmHg vs. 7.0 ± 2.7 mmHg, *p* < 0.001; PPV: 8.4 ± 3.5 mmHg vs. 7.6 ± 3.3 mmHg, *p* < 0.001). There was no significant difference in other variables between the two groups, including demographic and laboratory data (all *p* > 0.05).

**Table 1:** Baseline characteristics, visit-to-visit BP, BPV and outcome of the study participants.

Variable	Treatment arm		*p* value
	Standard treatment arm *N* = 4,176	Intensive treatment arm *N* = 4,170	
Female, *n* (%)	1,455 (34.8%)	1,471 (35.3%)	0.678
BMI (Kg/m^2^)	29.9 ± 5.7	30.0 ± 5.8	0.326
Age, year	67.7 ± 9.2	67.8 ± 9.2	0.878
Race, *n* (%)			0.224
Non-Hispanic White	2,441 (58.5%)	2,454 (58.8%)	
Non-Hispanic Black	1,252 (30.0%)	1,202 (28.8%)	
Hispanic	419 (10.0%)	428 (10.3%)	
Other	64 (1.5%)	86 (2.1%)	
Smoking status, *n* (%)			0.741
Never smoked	1,844 (44.2%)	1,836 (44.0%)	
Former smoker	1,796 (43.0%)	1,786 (42.8%)	
Current smoker	530 (12.7%)	545 (13.1%)	
Missing data	6 (0.1%)	3 (0.1%)	
Estimated GFR, mL min^−1^ 1.73 m^−2^, median (Q1–Q3)	71.4 (58.8–84.8)	71.6 (58.4–84.8)	0.878
Fasting total cholesterol, mg/dl, median (Q1–Q3)	187.0 (161.0–215.0)	186.0 (161.0–214.0)	0.996
Fasting total triglycerides, mg/dl, median (Q1–Q3)	107.0 (77.0–152.0)	107.0 (77.0–150.0)	0.984
Fasting HDL cholesterol, mg/dl, median (Q1–Q3)	50.0 (43.0–60.0)	50.0 (43.0–60.0)	0.913
Fasting glucose, mg/dl	97.0 (91.0–105.0)	97.0 (91.0–105.0)	0.948
Framingham 10-year CVD risk score, %, median (Q1–Q3)	17.8 (12.0–25.6)	17.6 (12.1–25.3)	0.702
Sub CVD	826 (19.8%)	829 (19.9%)	0.908
Times of blood pressure measurement	10.4 ± 2.3	11.2 ± 2.6	<0.001
Visit-to visit mean SBP, mmHg	135.3 ± 6.5	122.6 ± 7.3	<0.001
SBPV.ARV, mmHg	12.4 ± 5.2	11.5 ± 4.9	<0.001
Visit-to visit mean DBP, mmHg	75.1 ± 8.8	68.6 ± 7.9	<0.001
DBPV.ARV, mmHg	7.4 ± 2.8	7.0 ± 2.7	<0.001
Visit-to visit mean PP, mmHg	60.2 ± 10.2	54.0 ± 10.0	<0.001
PPV.ARV, mmHg	8.4 ± 3.5	7.6 ± 3.3	<0.001
**Outcome**			
MCI, *n* (%)	353 (8.4%)	285 (6.8%)	0.005
PD, *n* (%)	101 (2.4%)	75 (1.8%)	0.049

BP, blood pressure; BPV, blood pressure variability; SBPV, systolic blood pressure variability; DBPV, diastolic blood pressure variability; PPV, pulse pressure variability; BMI, body mass index; sub-CVD, subclinical cardiovascular disease; ARV, average real variability; MCI, mild cognitive impairment; PD, probable dementia.

In terms of outcome, the incidence of MCI and PD in the intensive group was lower than that in the standard group (MCI: 6.8% vs. 8.4%, *p* = 0.005; PD: 1.8% vs. 2.4%, *p* = 0.049).

As to the antihypertension (AH) medications, in the whole population, the average of the types of AH medications in the standard group was 1.9 ± 1.0 and patients in the intensive group took an average of 2.6 ± 1.0 types of AH medications. The types of AH medications for the different BPV tertiles were demonstrated in Supplementary Table S3.

### 3.2. Kaplan-Meier curves analysis among tertiles of BPV in different blood pressure treatment arms

**Figure 2:**
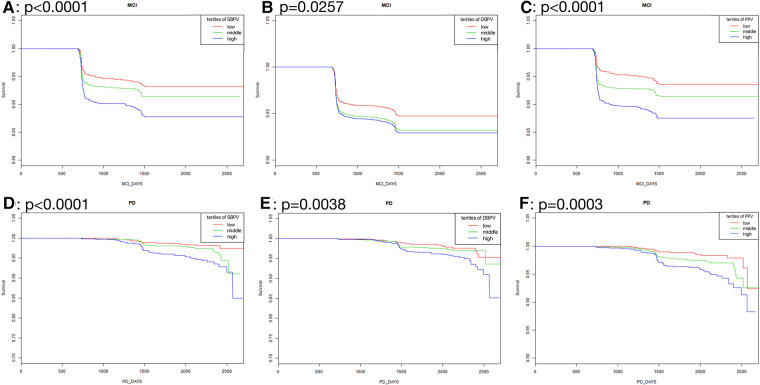
Kaplan-Meier curves analysis among tertiles of BPV in standard blood pressure treatment. (**A–C**) Showed the rates free of mild cognitive impairment in follow-up after stratification by SBPV, DBPV and PPV in standard blood pressure treatment; (**D–F**) showed the rates free of probable dementia in follow-up after stratification by SBPV, DBPV and PPV in standard blood pressure treatment.

**Figure 3:**
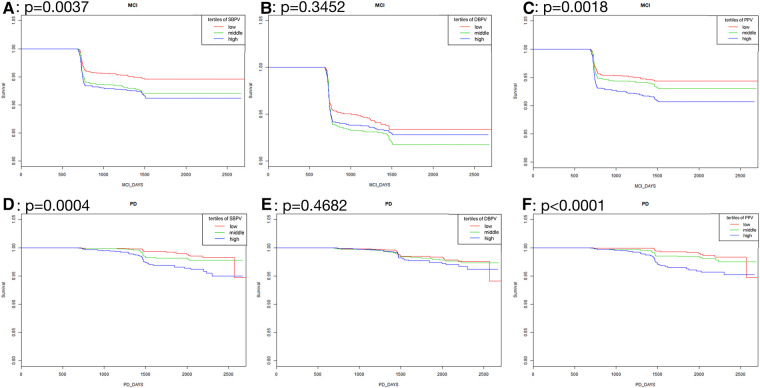
Kaplan-Meier curves analysis among tertiles of BPV in intensive blood pressure treatment. (**A–C**) Showed the rates free of mild cognitive impairment in follow-up after stratification by SBPV, DBPV and PPV in intensive blood pressure treatment; (**D–F**) showed the rates free of probable dementia in follow-up after stratification by SBPV, DBPV and PPV in intensive blood pressure treatment.

As was shown in Figure 2, in standard blood pressure treatment, tertiles with higher SBPV, DBPV, and PPV had a higher risk of MCI (SBPV: *p* < 0.0001; DBPV: *p* = 0.0257; PPV: *p* <0.0001) and PD (SBPV: *p* < 0.0001; DBPV: *p* = 0.0038; PPV: *p* =0.0003) respectively. As was shown in Figure 3, in intensive blood pressure treatment, tertiles with higher SBPV and PPV had a higher risk of MCI (SBPV: *p* = 0.0037; PPV: *p* = 0.0018) and PD (SBPV: *p* = 0.0004; PPV: *p* < 0.0001). However, there was no significant difference in the risk of MCI or PD among different DBPV tertiles (*p* = 0.3452 for MCI; *p* = 0.4682 for PD) in the intensive blood pressure treatment group.

### 3.3. Association between different BPV (SBPV, DBPV and PPV) and MCI in different blood pressure treatment arms

We used three COX regression models to analyze the association between BPV and MCI. As was shown in Table 2, in the standard group, the risks of MCI in T3 of SBPV and PPV were significantly higher than in T1 in Model 1 and Model 2. In Model 3, which considered visit-to-visit mean blood pressure, the risks of MCI in T3 of these three types were significantly higher than that in T1.(SBPV: HR(95%) = 1.7 (1.3–2.2), *p* < 0.001; DBPV: HR(95%) = 1.3 (1.0–1.7), *p* = 0.048; PPV: HR(95%) = 1.6 (1.2–2.2), *p* = 0.001).

**Table 2:** Association between BPV (SBPV, DBPV and PPV) and MCI in different blood pressure treatment arms.

BPV tertiles	Model 1	Model 2	Model 3
	HR (95%CI) *p* value		
**Standard treatment**			
SBPV.ARV			
T1	Ref.	Ref.	Ref.
T2	1.3 (0.9–1.7) 0.119	1.2 (0.9–1.7) 0.154	1.2 (0.9–1.7) 0.160
T3	1.7 (1.3–2.2) <0.001	1.7 (1.3–2.3) <0.001	1.7 (1.3–2.2) <0.001
DBPV.ARV			
T1	Ref.	Ref.	Ref.
T2	1.2 (0.9–1.5) 0.244	1.2 (0.9–1.6) 0.183	1.2 (0.9–1.6) 0.169
T3	1.3 (1.0–1.6) 0.082	1.3 (1.0–1.7) 0.058	1.3 (1.0–1.7) 0.048
PPV.ARV			
T1	Ref.	Ref.	Ref.
T2	1.4 (1.0–1.9) 0.043	1.4 (1.0–1.9) 0.033	1.4 (1.0–1.9) 0.039
T3	1.6 (1.2–2.2) <0.001	1.7 (1.3–2.3) <0.001	1.6 (1.2–2.2) 0.001
**Intensive treatment**			
SBPV.ARV			
T1	Ref.	Ref.	Ref.
T2	1.3 (1.0–1.6) 0.024	1.2 (1.0–1.5) 0.039	1.2 (1.0–1.5) 0.047
T3	1.5 (1.2–1.8) <0.001	1.5 (1.2–1.8) <0.001	1.4 (1.2–1.8) <0.001
DBPV.ARV			
T1	Ref.	Ref.	Ref.
T2	1.3 (1.0–1.7) 0.095	1.2 (0.9–1.7) 0.125	1.2 (0.9–1.6) 0.150
T3	1.2 (0.9–1.6) 0.184	1.2 (0.9–1.6) 0.312	1.1 (0.8–1.5) 0.439
PPV.ARV			
T1	Ref.	Ref.	Ref.
T2	1.0 (0.7–1.3) 0.761	0.9 (0.7–1.2) 0.533	0.9 (0.7–1.2) 0.497
T3	1.0 (0.7–1.3) 0.989	0.9 (0.7–1.2) 0.596	0.9 (0.7–1.2) 0.483

Model 1 was adjusted for age, body mass index, sex and race.

Model 2 was adjusted for age, body mass index, sex, race, smoke, estimated glomerular filtration rate, Framingham 10-year cardiovascular disease risk score, subclinical cardiovascular disease and total cholesterol.

Model 3 was adjusted for age, body mass index, sex, race, smoke, estimated glomerular filtration rate, Framingham 10-year cardiovascular disease risk score, subclinical cardiovascular disease, total cholesterol and visit-to-visit mean systolic blood pressure or diastolic blood pressure or pulse pressure.

In the intensive group, only the highest tertile of SBPV remained a significant difference in the risk of MCI compared with the lowest in Model 1 and Model 2 (SBPV: HR(95%) = 1.5 (1.2–1.8), *p* < 0.001 in Model 2; DBPV: HR(95%) = 1.2 (0.9–1.6), *p* = 0.312 in Model 2; PPV: HR(95%) = 0.9 (0.7–1.2), *p* = 0.596 in Model 2). The adjustment of visit-to-visit mean blood pressure in model 3 did not change the association between higher SBPV and the growing risk of MCI (HR(95%) = 1.4 (1.2–1.8), *p* < 0.001).

In the standard group, elevated BPV was significantly associated with an increased risk of MCI in Model 3. In the intensive group, only higher SBPV was statistically significant with the increased risk of MCI.

### 3.4. Association between different BPV (SBPV, DBPV and PPV) and Pd in different blood pressure treatment arms

We adjusted the same models for the association between different BPV and PD. As was shown in Table 3, in the standard group, the risk of PD in T3 of SBPV and DBPV were significantly higher than in T1 in Model 1 (SBPV: HR(95%) = 2.5 (1.4–4.4), *p* = 0.002; DBPV: HR(95%) = 2.0 (1.2–3.3), *p* = 0.008). In Model 2 with multiple covariates adjusted, the risks of MCI in T3 of SBPV and PPV were significantly higher than that in T1 (SBPV: HR(95%) = 2.4 (1.4–4.3), *p* = 0.003; PPV: HR(95%) = 1.8 (1.0–3.4), *p* = 0.049). In Model 3, which considered visit-to-visit mean blood pressure, the risks of PD in T3 of all these three types were significantly higher than in T1.(SBPV: HR(95%) = 2.4 (1.4–4.4), *p* = 0.003; DBPV: HR(95%) = 1.8 (1.1–3.0), *p* = 0.030; PPV: HR(95%) = 2.0 (1.1–3.7), *p* = 0.026).

**Table 3:** Association between BPV (SBPV, DBPV and PPV) and PD in different blood pressure treatment arms.

BPV tertiles	Model 1	Model 2	Model 3
	HR (95%CI) *p* value		
**Standard treatment**			
SBPV.ARV			
T1	Ref.	Ref.	Ref.
T2	1.6 (0.8–2.9) 0.170	1.4 (0.8–2.8) 0.261	1.4 (0.8–2.8) 0.259
T3	2.5 (1.4–4.4) 0.002	2.4 (1.4–4.3) 0.003	2.4 (1.4–4.4) 0.003
DBPV.ARV			
T1	Ref.	Ref.	Ref.
T2	1.1 (0.6–1.9) 0.717	1.1 (0.6–2.0) 0.639	1.1 (0.6–2.0) 0.725
T3	2.0 (1.2–3.3) 0.008	2.0 (1.2–3.3) 0.011	1.8 (1.1–3.0) 0.030
PPV.ARV			
T1	Ref.	Ref.	Ref.
T2	1.6 (0.9–3.0) 0.118	1.8 (0.9–3.4) 0.074	1.8 (1.0–3.5) 0.059
T3	1.8 (1.0–3.2) 0.053	1.8 (1.0–3.4) 0.049	2.0 (1.1–3.7) 0.026
**Intensive treatment**			
SBPV.ARV			
T1	Ref.	Ref.	Ref.
T2	1.5 (0.8–2.9) 0.188	1.4 (0.8–2.8) 0.270	1.4 (0.8–2.8) 0.265
T3	2.2 (1.2–4.1) 0.010	2.0 (1.1–3.8) 0.029	2.1 (1.1–3.9) 0.026
DBPV.ARV			
T1	Ref.	Ref.	Ref.
T2	1.1 (0.6–1.9) 0.796	1.0 (0.6–1.8) 0.915	1.0 (0.6–1.8) 0.896
T3	1.3 (0.7–2.2) 0.390	1.2 (0.7–2.1) 0.564	1.2 (0.7–2.2) 0.535
PPV.ARV			
T1	Ref.	Ref.	Ref.
T2	1.1 (0.6–2.1) 0.800	1.0 (0.5–2.0) 0.941	1.0 (0.5–2.0) 0.914
T3	2.2 (1.2–3.9) 0.012	2.0 (1.1–3.6) 0.030	2.0 (1.1–3.8) 0.025

Model 1 was adjusted for age, body mass index, sex and race.

Model 2 was adjusted for age, body mass index, sex, race, smoke, estimated glomerular filtration rate, Framingham 10-year cardiovascular disease risk score, subclinical cardiovascular disease and total cholesterol.

Model 3 was adjusted for age, body mass index, sex, race, smoke, estimated glomerular filtration rate, Framingham 10-year cardiovascular disease risk score, subclinical cardiovascular disease, total cholesterol and visit-to-visit mean systolic blood pressure or diastolic blood pressure or pulse pressure.

In the intensive group, the highest tertiles of SBPV and PPV, compared with the lowest, increased the risk of PD in Model 1. After adjusting the covariates in Model 2, the highest tertiles of SBPV and PPV remained a significant difference with the risk of MCI compared with the lowest (SBPV: HR(95%) = 2.0 (1.1–3.8), *p* = 0.029; DBPV: HR(95%) = 1.2 (0.7–2.1); *p* = 0.564; PPV: HR(95%) = 2.0 (1.1–3.6), *p* = 0.030). The adjustment of visit-to-visit mean blood pressure in model 3 did not change the association between higher SBPV or PPV and the growing risk of MCI (SBPV: HR(95%) = 2.1 (1.1–3.9), *p* = 0.026; PPV: HR(95%) =2.0 (1.1–3.8), *p* = 0.025).

In the standard group, elevated BPV was significantly associated with an increased risk of PD in Model 3. In the intensive group, higher SBPV and PPV were statistically significant with the increased risk of PD.

### 3.5. Interaction analysis for the risk of MCI or PD by BPV (SBPV, DBPV and PPV) tertiles

As was shown in Table 4, the interaction analysis was used in all three different BPV (SBPV, DBPV and PPV) types (all *p* for interaction >0.05). It demonstrated that compared with standard blood pressure treatment, intensive blood pressure treatment made no difference in the effect of the higher tertile of BPV on the risk of MCI and PD.

**Table 4:** Interaction analysis between different blood pressure treatment arms (standard blood pressure treatment vs. Intensive blood pressure treatment) on the risk of MCI or PD by BPV (SBPV, DBPV and PPV).

Blood pressure treatment arms	HR (95%CI)	*p* for interaction
**MCI**		
SBPV.ARV		0.6416
Standard treatment	1.0 (1.0–1.1) 0.0004	
Intensive treatment	1.0 (1.0–1.1) 0.0161	
DBPV.ARV		0.6521
Standard treatment	1.0 (1.0–1.1) 0.0483	
Intensive treatment	1.0 (1.0–1.1) 0.2628	
PPV.ARV		0.1478
Standard treatment	1.1 (1.0–1.1) <0.0001	
Intensive treatment	1.0 (1.0–1.1) 0.1637	
**PD**		
SBPV.ARV		0.9431
Standard treatment	1.1 (1.0–1.1) 0.0025	
Intensive treatment	1.1 (1.0–1.1) 0.0108	
DBPV.ARV		0.3962
Standard treatment	1.1 (1.0–1.2) 0.0021	
Intensive treatment	1.1 (1.0–1.2) 0.1699	
PPV.ARV		0.6881
Standard treatment	1.1 (1.0–1.1) 0.0275	
Intensive treatment	1.1 (1.0–1.1) 0.0113	

## 4. Discussion

In this *post hoc* analysis of the SPRINT MIND trial, we investigated the association of three types of BPV with MCI and PD in intensive and standard blood pressure treatment. In the standard group, we found that the increase of SBPV, DBPV and PPV showed a positive relationship with the higher risk of MCI and PD. In the intensive group, we found higher SBPV and PPV were associated with an increased risk of PD and that higher SBPV was associated with an increased risk of MCI. Moreover, the difference between intensive and standard blood pressure treatment was not statistically significant when we considered the effect of the higher BPV on the risk of MCI and PD. These findings emphasized the need for clinical work to monitor BPV in intensive blood pressure treatment.

In the standard group, previous studies assessed the different effects of three types of BPV on different domains of cognition. Tan Lai Zhou (21) evaluated the effect of higher SBPV and DBPV on cognitive impairment by memory function, information processing speed and executive function in 40- to 75-year-old individuals from The Maastricht Study. They did a further investigation into three different cognitive domains. They concluded that greater DBPV was associated with both lower information processing speed and executive function and was marginally associated with lower memory function. In contrast, greater SBPV was marginally associated with a lower memory function. In addition, after a cohort study of the association of SBPV and DBPV with the increased risk of incident dementia in 6,506 elderly individuals followed-up for 8 years, Annick Alpérovitch (22) presented that an increase of 1 standard deviation in the coefficient of SBPV or DBPV was associated with an increased risk of dementia of about 10%. However, Laure Rouch (7) showed that PPV was no longer associated with cognition function in a total of 3,319 subjects from the S.AGES cohort. The possible reason was that their participants were with chronic pain, type 2 diabetes mellitus or atrial fibrillation, which might affect the basic level of pulse pressure.

Moreover, this study further explored the relationship between various BPV and MCI or PD in intensive blood pressure treatment. Previous research has found that SBPV was associated with the development of probable dementia in intensive blood pressure treatment (14). Our study found that in the intensive group, the increase of SBPV significantly increased the risk of PD and MCI, and the increase of PPV significantly increased the risk of PD. However, our study did not find a relationship between DBPV and PD or MCI. There was a mechanism that could explain the different effects of SBPV, DBPV or PPV on the risk of MCI and PD in intensive blood pressure treatment. Smith EE (23) have shown that BPV could be responsible for cerebral small vessel disease and which was one of the possible mechanisms of MCI and PD (24). Meanwhile, white matter hyperintensities (WMH) represented the progression of cerebral small vessel disease (25) and Van Middelaar T (26) indicated that higher SBPV and PPV were associated with more progression of WMH while DBPV was not associated with WMH progression. Although the SPRINT MIND trial has shown that intensive blood pressure treatment was associated with slower progression of white matter hyperintensities (6), it did not change the difference in the association between three types of BPV and the progression of white matter hyperintensities. Therefore, according to the progression of WMH, there might be a strong association of SBPV or PPV with cerebral small vessel disease, while DBPV had no such correlation.

Compared with standard blood pressure treatment, we found that the strong relationship between higher BPV and increased risk of MCI and PD did not change after implementing intensive blood pressure treatment. Our findings were consistent with the previous study where Adam de Havenon (17) showed that SBPV remained a risk factor for developing cognitive impairment despite the excellent blood pressure treatment in the SPRINT MIND trial. We further investigated DBPV and PPV measured by ARV and found the relationship was consistent in all three types of BPV. Large numbers of previous studies that showed BPV was a risk factor for incident dementia independent of mean BP levels were based on standard blood pressure treatment (1, 8, 27). Our study made a supplement that BPV was still an independent risk factor for MCI and PD in intensive blood pressure treatment. Various studies have found different mechanisms to explain the independent effect of elevated BPV on the increased risk of MCI and PD. Firstly, higher BPV was associated with endothelial injury (28), and injury in cerebral microvessels may compromise the function of the blood-brain barrier and increase vascular permeability and protein extravasation in cerebral parenchyma (29). Secondly, BPV could be responsible for cerebral small vessel diseases, including white matter lesions, microbleeds, silent infarcts, and brain atrophy, resulting in MCI and PD (18, 30). Moreover, elevated short-term BPV is associated with lower cerebrovascular reactivity (CVR), independent of mean BP levels (31). CVR was a marker of cerebrovascular dysfunction or prodromal disease, which represented the ability of the brain's vessels to dilate and constrict in response to vasoactive stimuli (32).

The strengths of our study included the use of three types of BPV and the well-characterized, large study population, which made it possible to adjust for a large variety of covariates. The adjustment of visit-to-visit mean blood pressure in model 3 increased the credibility of the article.

We acknowledged that there were some limitations of our study. First of all, it was a *post hoc* analysis of a trial that conformed to neither the population nor the randomization model of statistical inference. Secondly, the trial excluded persons with type 2 diabetes, previous stroke, advanced kidney disease, or symptomatic heart failure which limited our results to patients without corresponding basic diseases. Thirdly, the assessment of cognitive function was based on the endpoint events (MCI or PD), which were unable to evaluate either different cognitive dimensions or subtypes of MCI and PD.

## 5. Conclusion

In this *post hoc* analysis of the SPRINT MIND trial, we found that, in standard blood pressure treatment, elevated BPV, regardless of using any of the three BPV types, was significantly associated with an increased risk of MCI. In intensive blood pressure treatment, higher SBPV and PPV were associated with an increased risk of PD, and higher SBPV was associated with an increased risk of MCI. The effect of higher BPV on the risk of MCI and PD was not significantly different in intensive and standard blood pressure treatment. These findings indicated visit-to-visit BPV as a risk factor for MCI and PD, independent of mean blood pressure. Moreover, it was of great value for clinical work to monitor BPV, especially SBPV or PPV, in both standard and intensive blood pressure treatment.

## Data Availability

Publicly available datasets were analyzed in this study. This data can be found here: the National Institutes of Health Biologic Specimen and Data Repository Information Coordinating Center at https://biolincc.nhlbi.nih.gov/studies/sprint.
