# Supplementary material for: Effect of visit-to-visit blood pressure variability on mild cognitive impairment and probable dementia in hypertensive patients receiving standard and intensive blood pressure treatment
Source: Front Cardiovasc Med. 2023 Apr 17;10:1166554. doi: 10.3389/fcvm.2023.1166554 (PMC10150011; doi:10.3389/fcvm.2023.1166554)
Supplement: Supplementary file 1 [file Table1.docx]

SD was calculated using the following formula:  SD=$\sqrt{(\frac{1}{n-1})\sum_{i=1}^{n} {({BP}_{i}-{BP}_{mean})}^{2}}$

**TableS1. Association between BPV (SBPV, DBPV and PPV, measured by SD) and MCI in different blood pressure treatment arms**

| BPV tertiles | Model 1 | | Model 2 | Model 3 |
| --- | --- | --- | --- | --- |
|  | HR (95%CI) P value | | | |
| Standard treatment |  | |  |  |
| SBPV.SD |  | |  |  |
| T1 | Ref. | | Ref. | Ref. |
| T2 | 1.2 (0.9-1.5) 0.279 | | 1.2 (0.9-1.5) 0.294 | 1.2 (0.9-1.5) 0.297 |
| T3 | 1.5 (1.2-2.0) 0.002 | | 1.6 (1.2-2.0) 0.001 | 1.5 (1.2-2.0) 0.002 |
| DBPV.SD |  | |  |  |
| T1 | Ref. | | Ref. | Ref. |
| T2 | 1.2 (0.9-1.5) 0.231 | | 1.2 (0.9-1.5) 0.179 | 1.2 (0.9-1.6) 0.164 |
| T3 | 1.2 (0.9-1.6) 0.159 | | 1.2 (0.9-1.6) 0.117 | 1.3 (1.0-1.6) 0.102 |
| PPV.SD |  | |  |  |
| T1 | Ref. | | Ref. | Ref. |
| T2 | 1.2 (0.9-1.6) 0.297 | | 1.2 (0.9-1.6) 0.244 | 1.2 (0.9-1.6) 0.268 |
| T3 | 1.6 (1.2-2.2) <0.001 | | 1.7 (1.3-2.2) <0.001 | 1.7 (1.2-2.2) <0.001 |
| Intensive treatment |  | |  |  |
| SBPV.SD |  | |  |  |
| T1 | Ref. | | Ref. | Ref. |
| T2 | 1.2 (0.9-1.4) 0.148 | | 1.1 (0.9-1.4) 0.188 | 1.1 (0.9-1.4) 0.214 |
| T3 | 1.3 (1.1-1.6) 0.004 | | 1.3 (1.1-1.6) 0.008 | 1.3 (1.0-1.6) 0.020 |
| DBPV.SD |  | |  |  |
| T1 | Ref. | | Ref. | Ref. |
| T2 | 1.5 (1.1-2.0) 0.006 | 1.5 (1.1-2.0) 0.008 | | 1.5 (1.1-2.0) 0.012 |
| T3 | 1.2 (0.9-1.6) 0.276 | 1.1 (0.8-1.5) 0.492 | | 1.1 (0.8-1.5) 0.620 |
| PPV.SD |  | |  |  |
| T1 | Ref. | | Ref. | Ref. |
| T2 | 1.1 (0.8-1.5) 0.490 | | 1.1 (0.8-1.5) 0.651 | 1.1 (0.8-1.5) 0.699 |
| T3 | 1.1 (0.8-1.5) 0.457 | | 1.0 (0.7-1.4) 0.927 | 1.0 (0.7-1.4) 0.941 |

Model 1 was adjusted for age, body mass index, sex and race.

Model 2 was adjusted for age, body mass index, sex, race, smoke, estimated glomerular filtration rate, Framingham 10-year cardiovascular disease risk score, subclinical cardiovascular disease and total cholesterol.

Model 3 was adjusted for age, body mass index, sex, race, smoke, estimated glomerular filtration rate, Framingham 10-year cardiovascular disease risk score, subclinical cardiovascular disease, total cholesterol and visit-to-visit mean systolic blood pressure or diastolic blood pressure or pulse pressure.

**TableS2. Association between BPV (SBPV, DBPV and PPV, measured by SD) and PD in different blood pressure treatment arms**

| BPV tertiles | Model 1 | | Model 2 | Model 3 |
| --- | --- | --- | --- | --- |
|  | HR (95%CI) P value | | | |
| Standard treatment |  | |  |  |
| SBPV.SD |  | |  |  |
| T1 | Ref. | | Ref. | Ref. |
| T2 | 1.8 (1.0-3.2) 0.034 | | 1.7 (1.0-3.1) 0.052 | 1.7 (1.0-3.1) 0.052 |
| T3 | 2.3 (1.3-3.9) 0.003 | | 2.2 (1.2-3.8) 0.006 | 2.2 (1.2-3.8) 0.007 |
| DBPV.SD |  | |  |  |
| T1 | Ref. | | Ref. | Ref. |
| T2 | 1.5 (0.9-2.5) 0.107 | | 1.6 (0.9-2.6) 0.093 | 1.5 (0.9-2.5) 0.137 |
| T3 | 1.9 (1.1-3.1) 0.016 | | 1.8 (1.1-3.0) 0.023 | 1.7 (1.0-2.8) 0.059 |
| PPV.SD |  | |  |  |
| T1 | Ref. | | Ref. | Ref. |
| T2 | 1.4 (0.8-2.5) 0.225 | | 1.5 (0.8-2.7) 0.167 | 1.6 (0.9-2.8) 0.129 |
| T3 | 1.8 (1.0-3.1) 0.035 | | 1.8 (1.0-3.2) 0.037 | 2.0 (1.1-3.6) 0.016 |
| Intensive treatment |  | |  |  |
| SBPV.SD |  | |  |  |
| T1 | Ref. | | Ref. | Ref. |
| T2 | 1.7 (1.1-2.7) 0.010 | | 1.7 (1.1-2.5) 0.020 | 1.7 (1.1-2.5) 0.020 |
| T3 | 1.9 (1.2-2.9) 0.003 | | 1.7 (1.1-2.7) 0.012 | 1.7 (1.1-2.7) 0.013 |
| DBPV.SD |  | |  |  |
| T1 | Ref. | | Ref. | Ref. |
| T2 | 0.8 (0.5-1.4) 0.446 | 0.7 (0.4-1.3) 0.307 | | 0.7 (0.4-1.3) 0.307 |
| T3 | 0.9 (0.5-1.5) 0.659 | 0.7 (0.4-1.3) 0.320 | | 0.7 (0.4-1.3) 0.323 |
| PPV.SD |  | |  |  |
| T1 | Ref. | | Ref. | Ref. |
| T2 | 1.4 (0.7-2.8) 0.328 | | 1.4 (0.7-2.7) 0.392 | 1.4 (0.7-2.8) 0.377 |
| T3 | 2.1 (1.1-4.0) 0.026 | | 1.8 (0.9-3.6) 0.075 | 1.9 (0.9-3.7) 0.070 |

Model 1 was adjusted for age, body mass index, sex and race.

Model 2 was adjusted for age, body mass index, sex, race, smoke, estimated glomerular filtration rate, Framingham 10-year cardiovascular disease risk score, subclinical cardiovascular disease and total cholesterol.

Model 3 was adjusted for age, body mass index, sex, race, smoke, estimated glomerular filtration rate, Framingham 10-year cardiovascular disease risk score, subclinical cardiovascular disease, total cholesterol and visit-to-visit mean systolic blood pressure or diastolic blood pressure or pulse pressure.

**TableS3. Association between BPV (SBPV, DBPV and PPV, measured by MMD) and MCI in different blood pressure treatment arms**

| BPV tertiles | Model 1 | | Model 2 | Model 3 |
| --- | --- | --- | --- | --- |
|  | HR (95%CI) P value | | | |
| Standard treatment |  | |  |  |
| SBPV.MMD |  | |  |  |
| T1 | Ref. | | Ref. | Ref. |
| T2 | 1.2 (0.9-1.6) 0.149 | | 1.2 (0.9-1.6) 0.158 | 1.2 (0.9-1.6) 0.169 |
| T3 | 1.6 (1.2-2.0) <0.001 | | 1.6 (1.2-2.1) <0.001 | 1.6 (1.2-2.1) 0.001 |
| DBPV.MMD |  | |  |  |
| T1 | Ref. | | Ref. | Ref. |
| T2 | 1.3 (1.0-1.7) 0.049 | | 1.3 (1.0-1.7) 0.069 | 1.3 (1.0-1.7) 0.065 |
| T3 | 1.2 (1.0-1.6) 0.105 | | 1.3 (1.0-1.6) 0.099 | 1.3 (1.0-1.7) 0.092 |
| Intensive treatment |  | |  |  |
| SBPV.MMD |  | |  |  |
| T1 | Ref. | | Ref. | Ref. |
| T2 | 1.1 (0.8-1.5) 0.524 | | 1.1 (0.8-1.5) 0.761 | 1.0 (0.7-1.4) 0.827 |
| T3 | 1.3 (1.0-1.8) 0.062 | | 1.2 (0.9-1.7) 0.217 | 1.2 (0.8-1.6) 0.329 |
| DBPV.MMD |  | |  |  |
| T1 | Ref. | | Ref. | Ref. |
| T2 | 1.1 (0.8-1.5) 0.559 | 1.1 (0.8-1.5) 0.635 | | 1.1 (0.8-1.4) 0.699 |
| T3 | 1.1 (0.8-1.4) 0.715 | 1.0 (0.7-1.3) 0.925 | | 1.0 (0.7-1.3) 0.775 |

Model 1 was adjusted for age, body mass index, sex and race.

Model 2 was adjusted for age, body mass index, sex, race, smoke, estimated glomerular filtration rate, Framingham 10-year cardiovascular disease risk score, subclinical cardiovascular disease and total cholesterol.

Model 3 was adjusted for age, body mass index, sex, race, smoke, estimated glomerular filtration rate, Framingham 10-year cardiovascular disease risk score, subclinical cardiovascular disease, total cholesterol and visit-to-visit mean systolic blood pressure or diastolic blood pressure or pulse pressure.

**TableS4. Association between BPV (SBPV, DBPV and PPV, measured by MMD) and PD in different blood pressure treatment arms**

| BPV tertiles | Model 1 | | Model 2 | Model 3 |
| --- | --- | --- | --- | --- |
|  | HR (95%CI) P value | | | |
| Standard treatment |  | |  |  |
| SBPV.MMD |  | |  |  |
| T1 | Ref. | | Ref. | Ref. |
| T2 | 1.2 (0.7-2.2) 0.432 | | 1.2 (0.7-2.1) 0.482 | 1.2 (0.7-2.1) 0.482 |
| T3 | 1.8 (1.1-3.1) 0.022 | | 1.7 (1.0-3.0) 0.038 | 1.7 (1.0-3.0) 0.040 |
| DBPV.MMD |  | |  |  |
| T1 | Ref. | | Ref. | Ref. |
| T2 | 1.7 (1.0-3.0) 0.039 | | 1.6 (0.9-2.7) 0.107 | 1.5 (0.9-2.5) 0.153 |
| T3 | 2.0 (1.2-3.4) 0.009 | | 1.8 (1.1-3.1) 0.026 | 1.7 (1.0-2.9) 0.061 |
| Intensive treatment |  | |  |  |
| SBPV.MMD |  | |  |  |
| T1 | Ref. | | Ref. | Ref. |
| T2 | 1.4 (0.7-2.6) 0.362 | | 1.2 (0.6-2.4) 0.533 | 1.2 (0.6-2.4) 0.531 |
| T3 | 1.5 (0.8-2.8) 0.196 | | 1.2 (0.6-2.4) 0.512 | 1.3 (0.6-2.5) 0.507 |
| DBPV.MMD |  | |  |  |
| T1 | Ref. | | Ref. | Ref. |
| T2 | 0.7 (0.4-1.3) 0.301 | 0.7 (0.4-1.3) 0.229 | | 0.7 (0.4-1.3) 0.229 |
| T3 | 0.9 (0.5-1.5) 0.613 | 0.7 (0.4-1.3) 0.289 | | 0.7 (0.4-1.3) 0.292 |

Model 1 was adjusted for age, body mass index, sex and race.

Model 2 was adjusted for age, body mass index, sex, race, smoke, estimated glomerular filtration rate, Framingham 10-year cardiovascular disease risk score, subclinical cardiovascular disease and total cholesterol.

Model 3 was adjusted for age, body mass index, sex, race, smoke, estimated glomerular filtration rate, Framingham 10-year cardiovascular disease risk score, subclinical cardiovascular disease, total cholesterol and visit-to-visit mean systolic blood pressure or diastolic blood pressure or pulse pressure.

TableS5. types of antihypertension medications for the BPV tertiles

| Variable | BPV tertiles | | | *P* value |
| --- | --- | --- | --- | --- |
|  | Low | Middle | High |  |
| Standard treatment |  |  |  |  |
| SBPV | 1.7 ± 1.0 | 1.9 ± 1.0 | 2.1 ± 1.0 | <0.001 |
| DBPV | 1.8 ± 1.0 | 1.9 ± 1.0 | 2.0 ± 1.0 | <0.001 |
| PPV | 1.7 ± 1.0 | 1.9 ± 1.0 | 2.0 ± 1.0 | <0.001 |
| Intensive treatment |  |  |  |  |
| SBPV | 2.5 ± 1.0 | 2.6 ± 1.0 | 2.7 ± 1.0 | <0.001 |
| DBPV | 2.5 ± 1.0 | 2.6 ± 1.0 | 2.6 ± 1.0 | 0.005 |
| PPV | 2.5 ± 1.0 | 2.6 ± 1.0 | 2.6 ± 1.0 | <0.001 |

**FigureS1: the times of BP measurements at baseline and every 100 days thereafter**

**FigureS2: the frequency of the BP measurements in all participants**
