# Supplementary figures and images for: Effect of visit-to-visit blood pressure variability on mild cognitive impairment and probable dementia in hypertensive patients receiving standard and intensive blood pressure treatment
Source: Front Cardiovasc Med. 2023 Apr 17;10:1166554. doi: 10.3389/fcvm.2023.1166554 (PMC10150011; doi:10.3389/fcvm.2023.1166554)

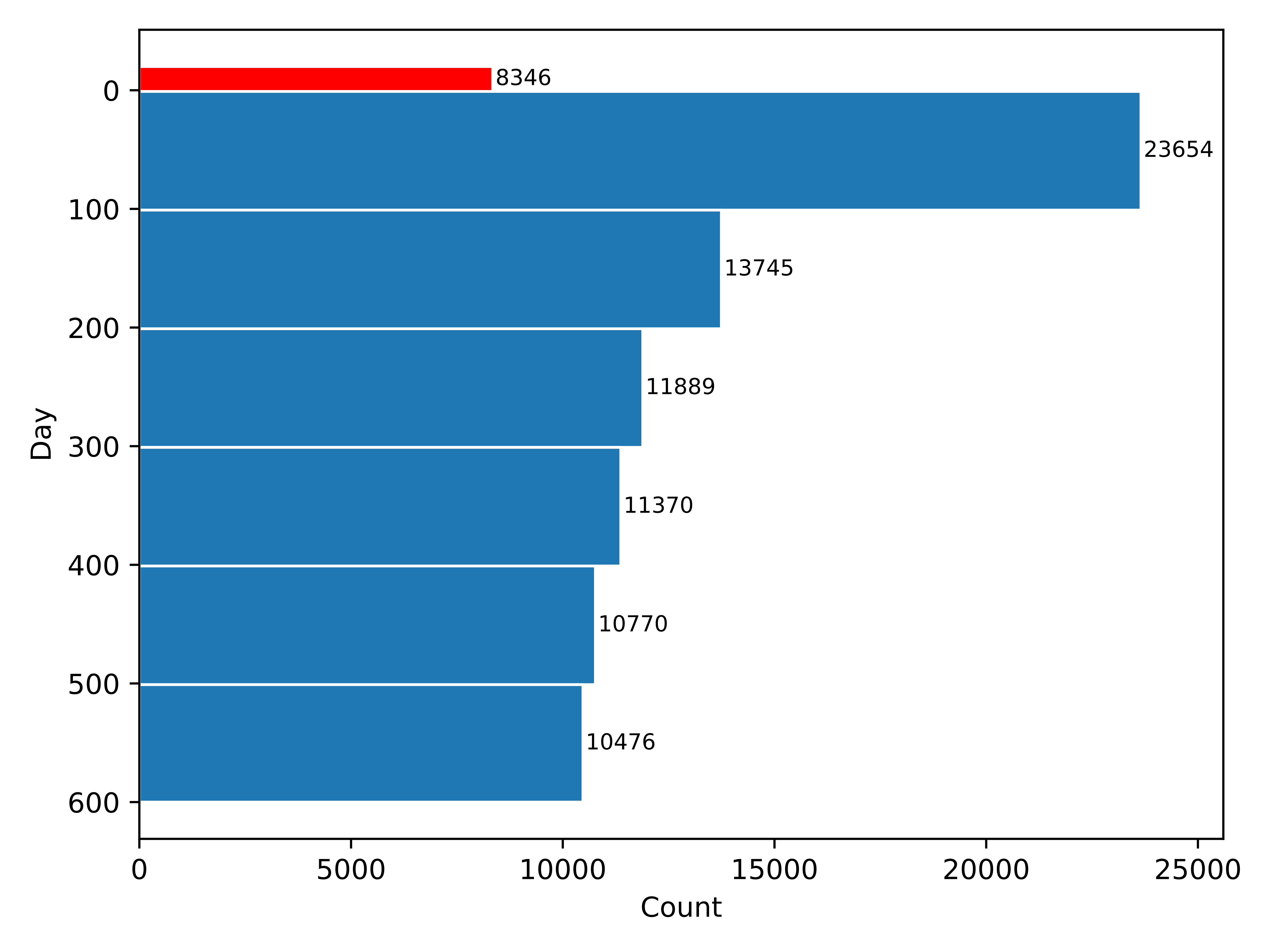

Supplement: Supplementary file 2 [file Image1.jpeg]

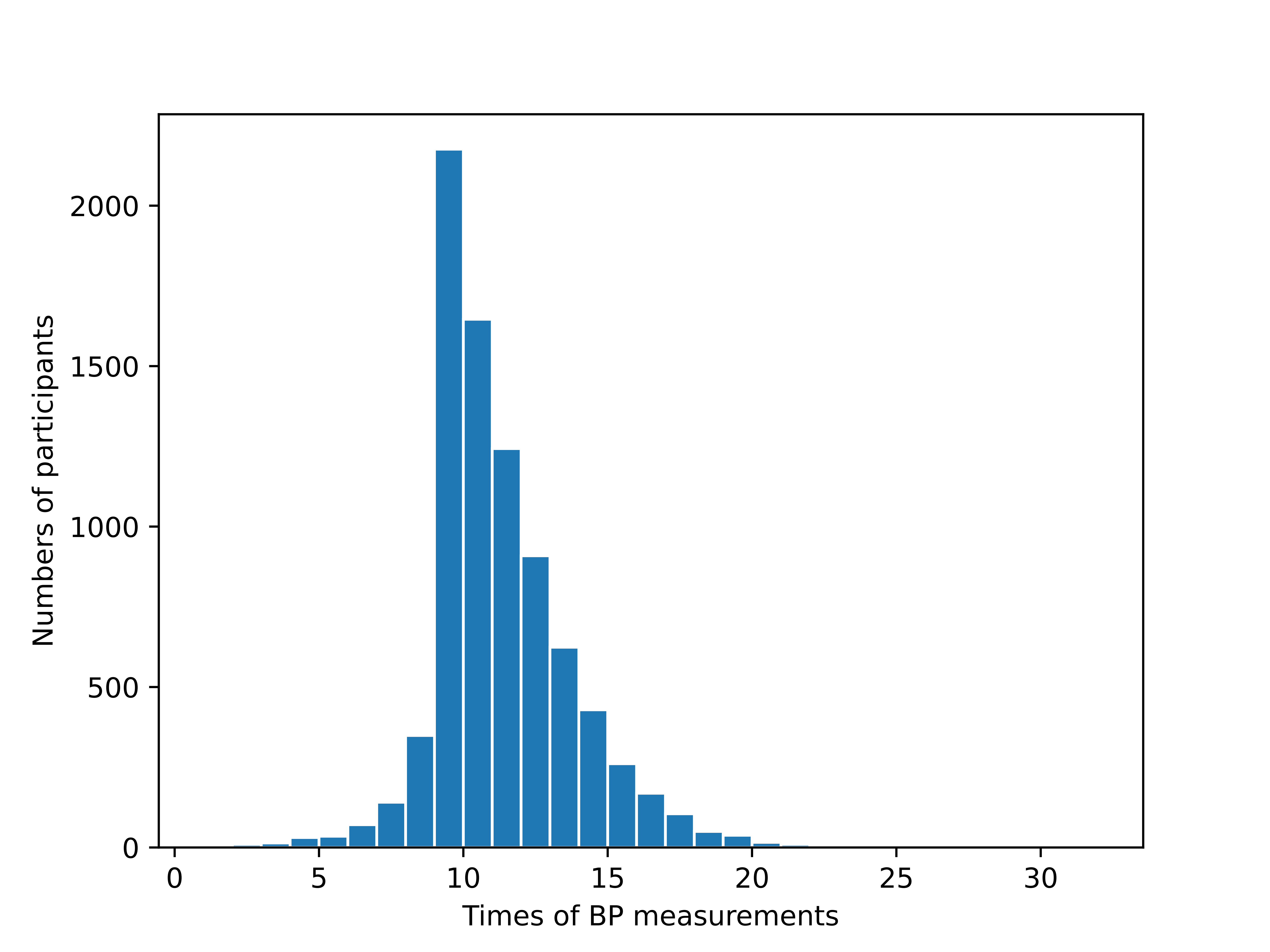

Supplement: Supplementary file 3 [file Image2.jpeg]
